# Intestinal fibrosis assessment in Crohn’s disease patient using unenhanced spectral CT combined with 3D-printing technique

**DOI:** 10.1186/s13244-025-01914-w

**Published:** 2025-03-20

**Authors:** Qiapeng Huang, Zhihui Chen, Ruonan Zhang, Huasong Cai, Xufeng Yang, Xiaodi Shen, Lili Huang, Xinyue Wang, Qingzhu Zheng, Mingzhe Li, Ziyin Ye, Xubin Liu, Ren Mao, Yangdi Wang, Jinjiang Lin, Zhoulei Li

**Affiliations:** 1 Department of Gastrointestinal Surgery, The First Affiliated Hospital, Sun Yat-Sen University, Guangzhou, People’s Republic of China; 2 Department of Radiology, The First Affiliated Hospital of Sun Yat-Sen University, Guangzhou, People’s Republic of China; 3 Center of Digestive Disease, The Seventh Affiliated Hospital of Sun Yat-Sen University, Shenzhen, People’s Republic of China; 4 Department of Pathology, The First Affiliated Hospital of Sun Yat-Sen University, Guangzhou, People’s Republic of China; 5 Department of Gastroenterology, The First Affiliated Hospital, Sun Yat-Sen University, Guangzhou, People’s Republic of China

**Keywords:** Crohn’s disease, Fibrosis, Computed tomography enterography, 3D printing

## Abstract

**Objectives:**

To integrate multiple parameters derived from unenhanced spectral CT with 3D-printing technique to accurately evaluate intestinal lesions in patients with Crohn’s disease (CD).

**Methods:**

Patients with proven CD who underwent preoperative spectral CT and surgery were included. The spectral CT images and histopathological specimens were achieved by employing 3D-printing technique. Diagnostic models were developed utilizing Z-Effective, Electron Density (ED), and Hounsfield unit (HU) values derived from spectral CT, along with spectral curve slopes λ_1_ and λ_2_, as well as ΔHU_MonoE_. The area under the receiver operating characteristic curve (AUC) and the influence of inflammation on the efficacy of the models were analyzed.

**Results:**

The ED and HU at MonoE 50 keV of the spectral CT were determined to exhibit the highest correlation with the fibrosis degree of the diseased intestine. The training dataset yielded an AUC of 0.828 (95% CI: 0.705–0.951). The sensitivity and specificity were calculated to be 77.3% and 82.6%, respectively. The AUC of the validation set was 0.812 (95% CI: 0.676–0.948) with a sensitivity of 63.6% and specificity of 89.7%. Moreover, our model demonstrated enhanced diagnostic accuracy for detecting fibrosis with an AUC value of 0.933 (95% CI: 0.856–1.000), sensitivity of 90.9%, and specificity of 87.0%, after regulating the influence of inflammation.

**Conclusion:**

The integration of unenhanced multi-parametric spectral CT and 3D-printing technique seems to be able to assess the intestinal fibrosis. Our diagnostic model remains effective in assessing the severity of fibrosis under presence of inflammation.

**Critical relevance statement:**

Our diagnostic model accurately assessed the degree of intestinal wall fibrosis in Crohn’s disease patients by using unenhanced spectral CT and 3D-printing technique, which could facilitate individualized treatment.

**Key Points:**

- Evaluating the extent of Crohn’s disease-related fibrosis is important.
- The combination of 3D-printing technique and multi-parametric spectral CT enhances diagnostic accuracy.
- The developed model using spectral CT allows for the assessment of intestinal fibrosis using multi-parameters.

**Graphical Abstract:**

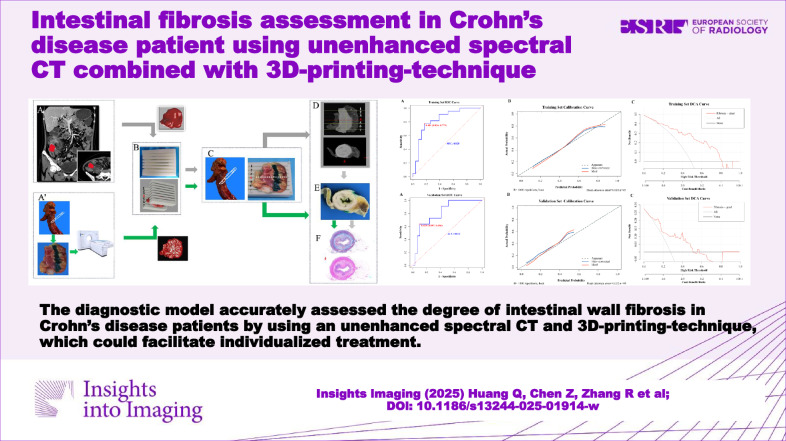

## Introduction

The development of intestinal stenosis due to fibrosis is a primary factor necessitating surgical intervention in patients with Crohn’s disease [1, 2]. Recurrences of intestinal strictures often require multiple bowel resections [3]. The prevalence of CD is highest in Western countries, while it is increasing in developing regions like Asia-Pacific [4–6]. Accurate qualitative and quantitative assessment of fibrotic involvement within the intestinal tract is anticipated to provide a precise foundation for surgery, effectively reducing unnecessary surgeries and enhancing patient prognosis, and even the quality of life [7, 8]. However, there is currently no clinically recognized method as standard for evaluating intestinal fibrosis [2, 9].

In general, the comprehensive evaluation of CD necessitates the integration of biochemical, endoscopic, pathological, and cross-sectional imaging data [10]. Although endoscopic pathology is a commonly employed method, it is both invasive and burdensome for CD patients with fibrous stenosis who may not tolerate additional biopsies [11]. Furthermore, a limitation lies in that endoscopy-obtained specimens can only observe mucosal and submucosal pathology but fail to detect pathology from the deep small intestine and deep intestinal wall [12].

In recent years, cross-sectional imaging, particularly computed tomography enterography (CTE), has played a pivotal role in CD diagnosis and treatment [13–15]. Its primary purpose is to assess the cumulative extent of intestinal wall lesions, lesion characteristics, and extraintestinal complications [16, 17]. With advancements in spectral CT, also known as dual-energy computed tomography, and the implementation of multi-parameter evaluation (virtual monochromatic images, iodine density, energy spectrum curve), the accuracy of CD diagnosis and quantification of disease activity can be significantly improved, which have provided a foundation for precise inflammation treatment [18–22]. It becomes even more crucial to quantify the status and extent of intestinal fibrosis. Currently, there is limited research on new technical parameters obtained from spectral CT for diagnosing CD intestinal fibrosis [23]. However, there is also a lack of comprehensive analysis regarding full-thickness binding pathology within the intestinal wall. Additionally, owing to the substantial heterogeneity of intestinal fibrosis, the monolayer imaging or pathological findings fail to accurately depict the true state of intestinal pathology.

Therefore, regarding to surgical specimens from CD patients, we designed a 3D printing box to obtain panoramic pathological sections of the intestinal wall with layer-to-layer correspondence to spectral CTE images. A multivariable logistic regression prediction model for tissue fibrosis degree was established by combining spectral CT and 3D-printing technique.

## Methods

### Patients

The present study was granted approval by the institutional ethics review board. A total of twelve CD patients who underwent spectral CTE and subsequently had surgery between January 20, 2023, and May 31, 2024, were enrolled in this study. Among them, the retrospective component comprised data from six CD patients as the experimental group, while an additional six cases were prospectively included as validation sets.

The inclusion criteria were as follows: (1) patients with a clear diagnosis of CD and intestinal fibrous stenosis complicated by standard clinical, endoscopic, radiographic, and histopathological criteria; (2) age ≥ 18 years old; (3) patients were diagnosed with symptomatic ileocolonic stenosis via spectral CTE imaging; (4) identification of the affected intestinal segment in spectral CTE imaging and obtaining histopathological specimens of the resected segment at the corresponding location. Exclusion criteria were: (1) poor imaging quality in spectral CTE examinations; (2) patients who underwent surgery more than 7 days after completing imaging examinations.

### Spectral CTE imaging and imaging analysis

The CTE protocol was outlined in our previous study (Supplementary Material 1 and Supplementary Tables 1, 2) [24]. Namely, Spectral CT (Philips Healthcare, Nederland B.V.) was used for CTE scanning. The tube voltage was 120 kVp, the slice thickness was 1.0 mm, and the reconstruction interval was 0.8 mm. The DoseRight automatic tube current modulation was employed to perform three phases of scanning: non-contrast, arterial, and venous phases. Subsequently, the spectral-based image (SBI) of the non-contrast phase was reconstructed.

To minimize bias, the target segment on the spectral CTE image was assessed by three experienced diagnostic radiologists (with 10 to 15 years of experience in abdominal imaging), who independently and simultaneously mapped the region of interest (ROI) to measure Z-Effective, Electron Density (ED), and HU at MonoE 40–140 keV. The energy spectrum curve-slope λ_1_, λ_2_ and ΔHU_MonoE_ in these regions were calculated using followed formula: λ_1_ = (HU_MonoE40keV_-HU_MonoE70keV_)/30, λ_2_ = (HU_MonoE40keV_-HU_MonoE140keV_)/100 and ΔHU_MonoE_ = HU_MonoE40keV_-HU_MonoE140keV_, respectively. Among them, HU_MonoE40keV_, HU_MonoE70keV_, and HU_MonoE140keV_ represented the CT values corresponding to the ROI at 40keV, 70keV, and 140keV, respectively. To minimize individual variations, was ROI plotted three times for each layer. The average of λ_1_, λ_2_, and ΔHU_MonoE_ from these three ROIs were then recorded.

### Layer-to-layer co-registration between CTE and specimen preparation with 3D-printing intestinal mold

**Fig. 1:**
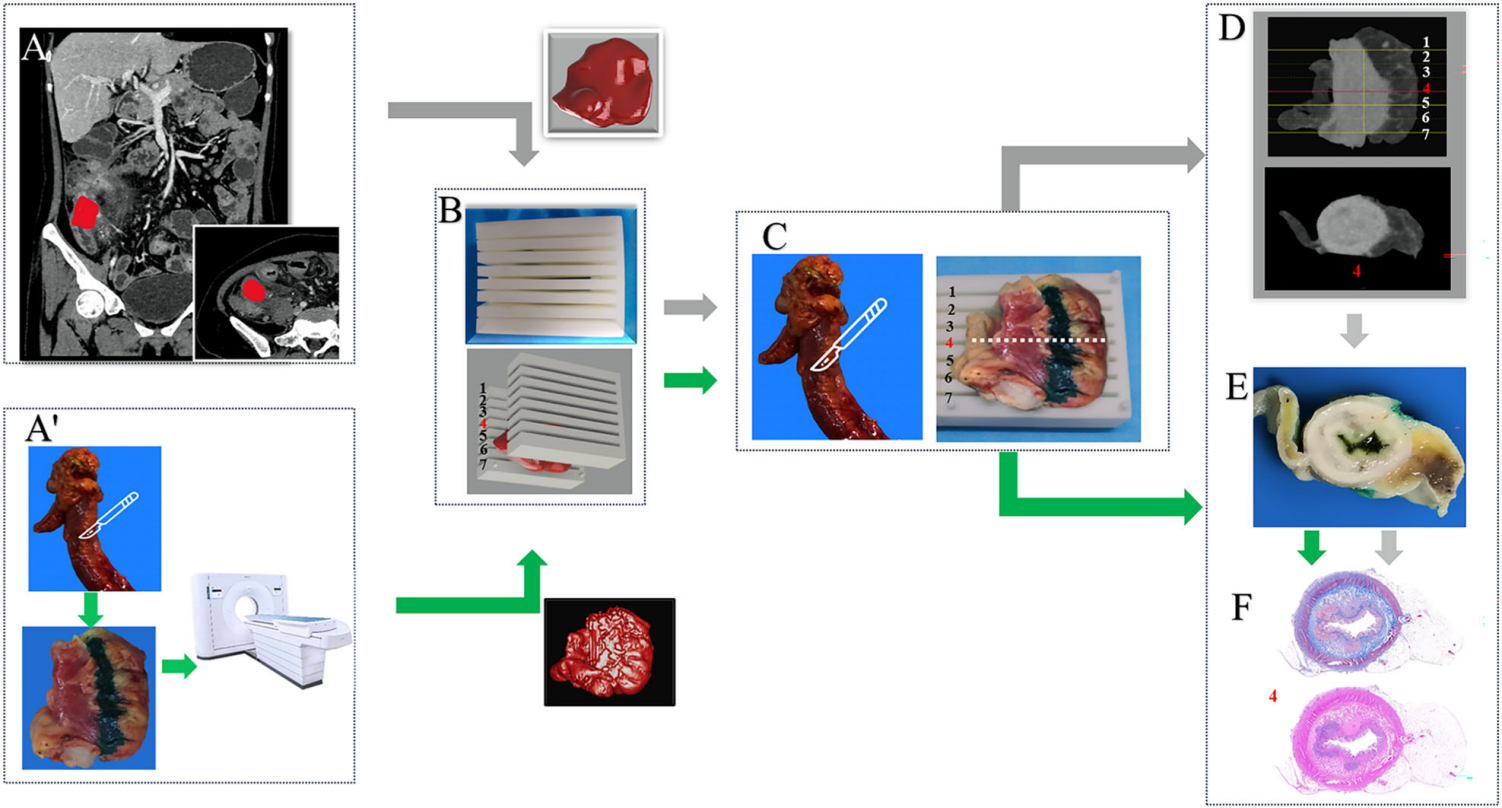
Longitudinal co-registration of CTE with intestinal specimens and pathological sections. **A’** An ex vivo spectral CT scan was performed on the resected intestine with stricture to obtain surface localization information, such as the middle layer. **A** The targeted intestinal stricture on the terminal ileum (indicated by the red zone) is outlined in the venous phase image for validation set. **B** A three-dimensional model is sketched to depict the target intestinal segment, based on which a 3D-printing mold is designed to accommodate the corresponding intestinal specimens. The mold incorporates grooves with a width of 2 mm and a spacing of 3 mm, while the number of slots is determined by the length of the target intestine. Specifically, the grooves are positioned with a center-to-center spacing of 5 mm, and the quantity of grooves is determined by the length of the target intestine (**B** has a total of 7 grooves, with the 4th groove located in the center, dividing the intestinal segment specimen into equal parts with a spacing of 5 mm between each part.). Subsequently, the digital model is transformed into a physical object through the utilization of 3D-printing technique. **C** According to the information obtained in CTE images of **A** and **A’**, meticulous removal of peristaltic fat surrounding the target intestinal segment was performed, followed by staining and marking with green for upper and lower boundaries. Then, the intestinal specimens were precisely positioned within the 3D-printing mold based on in vivo and ex vivo information. **D** An ex vivo CT scan was conducted on the specimen to acquire surface localization information such as the middle layer for validation set. For example, lines 1–7 on the external CT image corresponded to slots 1–7 on the mold depicted in **C**, while highlighting that red line 4 indicates the middle level (white dotted line in **C**). **E** The specimen is sliced at specified intervals to obtain complete circular sections of the intestinal axis, which correspond to the CTE images. **F** The intestinal sections are stained with Masson trichrome and Hematoxylin-eosin, digitally scanned to obtain electronic whole-circle whole-slide images for assessment. The workflow of longitudinal co-registration for training set was shown following green arrows, and for validation set was shown following gray ones

Three radiologists collaborated with a senior gastrointestinal surgeon to accurately determine the extent of resected terminal ileum, ranging from the level of the ileocecal valve to its proximal end. Key anatomical landmarks, such as the ileocecal valve, narrowest layer, and the length of targeted intestine, were accurately identified on CTE images. Referred to a previously reported study on 3D-printing technique [25, 26], CTE images were utilized to define an iso-surface for generating a 3D-printing mold to each resected targeted intestine in the validation set (Supplementary Material 1, 2). In the training set, the 3D-printing mold was designed directly based on the spectral CT imaging from the resected intestines, which is detailed in Fig. 1 and Supplementary Material 1, 2.

The surgeon performed a meticulous resection of the terminal ileum, followed by an immediate CT scan conducted by two radiologists to obtain crucial information such as the middle layer for precise surface localization. Subsequently, based on the orientation provided by CTE, the intestinal segment was meticulously restored to its original configuration and securely placed within a 3D-printed mold regarding previously obtained positioning marks. Finally, precise sectioning along designated slots allowed for obtaining axial whole-circle specimens corresponding to the CTE (Fig. 1 and Supplementary Material 2).

### Histopathological fibrotic assessment on whole-slide images

The intestinal tissues were first fixed in formalin. The axial whole-circle full-thickness specimens were then embedded in paraffin and sectioned into 4-µm-thick slices. Subsequently, Masson trichrome and Hematoxylin-eosin staining were performed, which were then digitally scanned to obtain electronic whole-circle whole-slide images for histopathological evaluation. In accordance with CTE assessment, two experienced pathologists who had no access to clinical or imaging information collectively scored the fibrotic or inflammatory severity using a previously described semi-quantitative scoring system ranging from 0 to 4 (Supplementary Tables 3, 4) [27].

### Statistical analysis

The statistical analyses were conducted using SPSS version 26.0 software (IBM SPSS Statistics 26. Ink), Python, and R software (version 4.2.3). In the subsequent multivariable logistic model analysis, the imaging parameters associated with fibrosis identified through lasso analysis were included. A dynamic nomogram of the model was constructed to supply a convenient and free using method for diagnosis of intestinal fibrosis. The differentiation ability and calibration performance of the dynamic nomogram of the model were evaluated by generating calibration curves, DCA diagrams, and receiver operating characteristic (ROC) curves. During the validation model, we calculated the area under the ROC curve (AUC) value for intestinal fibrosis diagnosis. Bootstrap method has been applied to improve the stability of the findings and reduces the potential impact of random error. Finally, by employing random seeds, we randomly screened imaging parameters of the affected intestinal segment while ensuring reproducibility to simulate the situation without utilizing 3D-printing technique. We then compared the diagnostic performance of fibrosis between 3D printing and none-3D printing conditions. Please refer to Supplementary Material 3 for more details.

## Results

### Demographic and clinical data

Twelve patients (6 men, 6 women; Mean age, 30.33 ± 7.99 years) were initially recruited in this study. A total of 85 segments, ranging from 4 to 13 sections per resected terminal ileum per patient, were obtained using 3D-printing technique. All 85 segments came from the narrowest region of the ileocecum. Their demographic and clinical characteristics are shown in Table 1.

**Table 1:** Baseline demographic and clinical characteristics of the 12 patients

Characteristic	Datum
Sex^a^	
Male	6/12 (50%)
Female	6/12 (50%)
Age (years)^b^	30.33 ± 7.99
BMI (kg/m^2^)^b^	17.40 ± 3.03
Disease duration (months)^c^	30.00 (11.50–102.00)
The interval between CTE imaging and surgery (days)^a^	
< 7	12/12 (100.0)
Surgery type^a^	
Ileocolon resection	9/12 (75.0)
Ileocolon + partial small bowel resection	3/12 (25.0)
Montreal location (L)^a^	
L3 (ilealcolonic)	11/12 (91.7)
L4 (upper gastrointestinal ± L1, L2, or L3)	1/12 (8.3)
No. of bowel specimens (*n* = 85)^a^	
Ileocecum	85/85 (100.0)
CDAI^b^	272.43 ± 128.83
CRP (mg/L)^b^	10.81 ± 9.33
ESR (mm/h)^b^	31.17 ± 20.83

*M* male, *F* female, *BMI* body mass index, *CTE* CT enterography, *CDAI* Crohn’s disease activity index, *CRP* C-reactive protein, *ESR* erythrocyte sedimentation rate

^a^ Categorical variables, expressed as frequencies (proportions)

^b^ Normally distributed continuous variables, expressed as mean ± standard deviation

^c^ Nonnormally distributed continuous variables, expressed as median (interquartile range)

### Histological results

The diseased intestinal wall, with a total of 85 segments, was classified into four categories based on the degree of fibrosis under score from 0 to 4 (Supplementary Table 3). Similarly, lesions were categorized with 4 degrees based on the severity of inflammation observed in the same segments (Supplementary Table 3). It is worth noting that higher degrees of inflammation and fibrosis are often associated with an increased risk of intestinal stricture as well as surgery [28–30]. To better identify patients at risk for surgery, we defined the none-mild fibrosis group (fibrosis score: 0 to 2) and moderate-severe fibrosis group (fibrosis score: 3 to 4). Similarly, we defined the none-mild (inflammatory score: 0 to 2) and moderate-severe inflammatory group (inflammatory score: 3 to 4) (Supplementary Tables 3, 4) [31]. Please refer to Supplementary Material 4 for more details.

### The spectral CT multi-parameters exhibited a distinct correlation with the pathological scores of intestinal fibrosis

The Shapiro–Wilk test result of each parameter showed that ED and HU_MonoE50–140keV_ followed a normal distribution (*p* > 0.05), while other parameters did not (*p* < 0.05) (Table 2). Based on this, Spearman’s rank correlation was used to analyze the correlation between Z-Effective, ED, λ_1_, λ_2_, ΔHU_MonoE_ and HU_MonoE40–140keV_ with fibrosis scores, where only ED and HU_MonoE40–140keV_ showed significant correlation with fibrosis scores (*p* < 0.001). After controlling inflammation as a covariate, the result of partial correlation analysis demonstrated that although there were changes in correlation coefficients between each parameter and pathological results of fibrosis, no statistical significance was observed (Table 3 and Supplementary Table 5).

**Table 2:** Normality test results of each parameter from the training set

Parameters	S-W	*p*-value
Z-Effective	0.737	< 0.001*
ED	0.905	0.231
λ_1_	0.967	0.024*
λ_2_	0.941	0.025*
ΔHU_MonoE_	0.942	0.025*
HU_MonoE40keV_	0.947	0.038*
HU_MonoE50keV_	0.960	0.127
HU_MonoE60keV_	0.971	0.327
HU_MonoE70keV_	0.976	0.471
HU_MonoE80keV_	0.980	0.623
HU_MonoE90keV_	0.982	0.710
HU_MonoE100keV_	0.983	0.756
HU_MonoE110keV_	0.984	0.789
HU_MonoE120keV_	0.985	0.801
HU_MonoE130keV_	0.985	0.804
HU_MonoE140keV_	0.985	0.810

According to the Shapiro–Wilk test, ED and HU_MonoE50–140keV_ are normally distributed (*p* > 0.05), while other parameters do not follow normal distribution (*p* < 0.05)

*S-W* Shapiro–Wilk Test, *ED* electron density, *HU* Hounsfield unit

* *p* < 0.05; λ, energy spectrum curve-slope; λ_1_, (HU_MonoE40keV_-HU_MonoE70keV_)/30; λ_2_, (HU_MonoE40keV_ -HU_MonoE140keV_)/100, ΔHU_MonoE_ = HU_MonoE40keV_-HU_MonoE140keV_

**Table 3:** Correlation analysis results and partial correlation analysis results between imaging parameters and fibrosis score

Parameters	*r*	*p-*value	*r*^a^	*p*-value^a^
Z-Effective	0.096	0.531	0.046	0.769
ED	0.535	< 0.001*	0.605	< 0.001*
λ_1_	0.086	0.575	0.005	0.975
λ_2_	0.088	0.565	0.003	0.983
ΔHU_MonoE_	0.089	0.561	0.003	0.983
HU_MonoE40keV_	0.484	< 0.001*	0.475	< 0.001*
HU_MonoE50keV_	0.485	< 0.001*	0.524	< 0.001*
HU_MonoE60keV_	0.540	< 0.001*	0.550	< 0.001*
HU_MonoE70keV_	0.534	< 0.001*	0.563	< 0.001*
HU_MonoE80keV_	0.529	< 0.001*	0.570	< 0.001*
HU_MonoE90keV_	0.527	< 0.001*	0.574	< 0.001*
HU_MonoE100keV_	0.531	< 0.001*	0.576	< 0.001*
HU_MonoE110keV_	0.537	< 0.001*	0.578	< 0.001*
HU_MonoE120keV_	0.540	< 0.001*	0.578	< 0.001*
HU_MonoE130keV_	0.540	< 0.001*	0.580	< 0.001*
HU_MonoE140keV_	0.534	< 0.001*	0.580	< 0.001*

*r* and *p*-values are the correlation analysis results between each parameter and the lesion fibrosis score, there is a significant correlation between ED, HU_MonoE40–140keV_ and fibrosis score (*p* < 0.001); *r*^a^ and *p*^a^ value are the partial correlation analysis results between each parameter and the lesion fibrosis score. The partial correlation coefficients of ED, HU_MonoE50–140 keV_ are larger than the correlation coefficients, which indicate that after regulating the effect of intestinal inflammation, the parameters can better characterize the degree of intestinal fibrosis

*ED* electron density, *HU* hounsfield unit

* *p* < 0.05; *r*, the correlation coefficient between the imaging parameters and the fibrosis score of the diseased intestinal wall; *p*-value, *p*-value of correlation analysis between imaging parameters and fibrosis score; *r*^a^, partial correlation coefficient between imaging parameters and the fibrosis score of the lesion wall; *p*^a^ value, *p*-value of partial correlation analysis between imaging parameters and fibrosis score; λ, energy spectrum curve-slope; λ_1_, (HU_MonoE40keV_-HU_MonoE70keV_)/30; λ_2_, (HU_MonoE40keV_ -HU_MonoE140keV_)/100; ΔHU_MonoE_ = HU_MonoE40keV_ -HU_MonoE140keV_

Notably, a subsequent analysis of the differences between the groups showed both ED and HU_MonoE40–140keV_ exhibited a statistically significant distinction (*p* < 0.001) between the none-mild fibrosis and moderate-severe fibrosis groups (Table 4). Furthermore, with regard to data distribution, there were also discernible variations observed in the values of ED and HU_MonoE40–140keV_ among different groups (Supplementary Fig. 1).

**Table 4:** Analysis of difference between None-mild fibrosis and Moderate-severe fibrosis

Parameters	None-mild fibrosis	Moderate-severe fibrosis	*z/t*	*p*-value
Z-Effective^a^	7.043 (6.983–7.075)	7.056 (7.011–7.072)	0.988	0.323
ED^b^	1.045 ± 0.010	1.056 ± 0.007	4.128	< 0.001*
λ_1_^a^	0.693 (0.587–0.833)	0.647 (0.597–0.786)	0.954	0.340
λ_2_^a^	0.278 (0.236–0.334)	0.260 (0.239–0.315)	0.976	0.329
ΔHU_MonoE_^a^	27.801 (23.547–33.409)	25.946 (23.901–31.504)	0.976	0.329
HU_MonoE40keV_^a^	11.883 (7.167–22.981)	29.223 (20.736–32.511)	3.519	< 0.001*
HU_MonoE50keV_^b^	25.553 ± 10.897	37.132 ± 7.697	−4.100	< 0.001*
HU_MonoE60keV_^b^	32.141 ± 10.316	43.370 ± 7.336	4.191	< 0.001*
HU_MonoE70keV_^b^	36.065 ± 10.042	47.086 ± 7.211	4.212	< 0.001*
HU_MonoE80keV_^b^	38.585 ± 9.896	49.471 ± 7.173	4.209	< 0.001*
HU_MonoE90keV_^b^	40.210 ± 9.839	51.017 ± 7.139	4.201	< 0.001*
HU_MonoE100keV_^b^	41.312 ± 9.790	52.058 ± 7.128	4.193	< 0.001*
HU_MonoE110keV_^b^	42.080 ± 9.753	52.792 ± 7.123	4.191	< 0.001*
HU_MonoE120keV_^b^	42.651 ± 9.748	53.317 ± 7.136	4.172	< 0.001*
HU_MonoE130keV_^b^	43.063 ± 9.717	53.726 ± 7.131	4.181	< 0.001*
HU_MonoE140keV_^b^	43.397 ± 9.717	54.012 ± 7.148	4.159	< 0.001*

There are statistically significant differences in ED and HU_MonoE40–140keV_ between the two groups (*p* < 0.001)

*ED* electron density, *HU* hounsfield unit

* *p* < 0.05; *z*, test statistics for Mann–Whitney U test; *t*, test statistics for two independent samples *T*-test; λ, energy spectrum curve-slope; λ_1_, (HU_MonoE40keV_-HU_MonoE70keV_)/30; λ_2_, (HU_MonoE40keV_ -HU_MonoE140keV_)/100; ΔHU_MonoE_ = HU_MonoE40keV_ - HU_MonoE140keV_

^a^ Nonnormally distributed continuous variables, expressed as median (interquartile range), line Mann–Whitney U test

^b^ Normally distributed continuous variables, expressed as mean ± standard deviation, line independent *T*-test

### Development of the diagnostic model for intestinal fibrosis and its efficacy evaluation

**Fig. 2:**
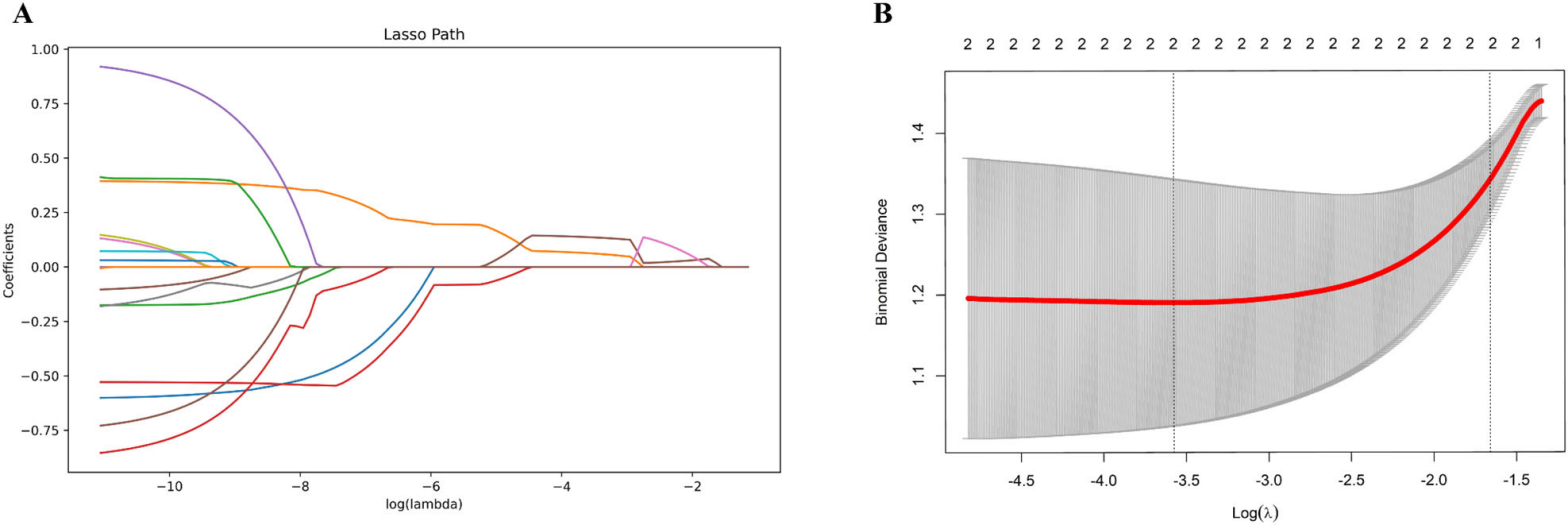
LASSO regression analysis of spectral CTE parameters. **A** The identification of the optimal penalty coefficient is achieved by 10-fold cross-validation and minimum criteria. The vertical line on the left side of (**B**) the λ_min_. Under this lambda value, the deviation is the smallest and the model fitting effect is the highest. Two variables (ED, HU_MonoE50keV_) are included in the equation. The right vertical line is λ_-se_, which represents the binomial deviation of cross-validation within 1 standard error to the right of the minimum λ

After conducting a preliminary analysis of the data characteristics, we employed LASSO regression analysis to screen the 16 included spectral CTE parameters. Eventually, we identified two factors that were most relevant to the fibrosis score group: ED and HU_MonoE50keV_. The process is illustrated in Fig. 2. The LASSO regression screening process adhered to the prescribed formula:

$$J(\beta )=\sum {(y-X\beta )}^{2}+\lambda {\parallel \beta \parallel }_{1}$$

Please refer to Supplementary Material 5 for more details.

Subsequently, we established a web-based dynamic nomogram accessible at https://dynanomo.shinyapps.io/Fibrosis_Prediction/ (Supplementary Fig. 2B). Please refer to Supplementary Material 6 for more details.

**Fig. 3:**
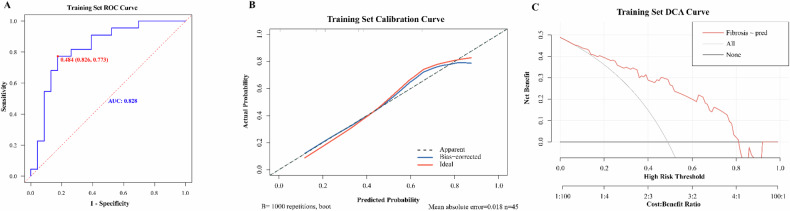
Analysis of the performance of fibrosis diagnostic model in training set. **A** The ROC curve for the training set data. The model group’s nomogram prediction model has a high accuracy in distinguishing between None-mild and Moderate-severe intestinal wall fibrosis in patients with Crohn’s disease. The AUC is 0.828 (95% confidence interval: [0.705, 0.951], sensitivity is 77.3%, specificity is 82.6%) and the C-index is 0.828. **B** The ratio between the average diagnostic probability of the nomogram prediction model based on ED and HU_MonoE50keV_ in the training set. A locally weighted regression line is plotted to show the overall trend. The red line indicates the ideal calibration curve. The calibration curve shows that the model has a good agreement between the prediction of the degree of intestinal wall fibrosis in the patients with Crohn’s disease and the actual pathological results. **C** The DCA curve of nomogram in training set. The *Y*-axis represents the net benefit, and the *X*-axis represents the high-risk threshold for predicting fibrosis selected between 0 and 1. The curve shows that the model provides a high benefit value for Crohn’s patients predicting diseased intestinal wall fibrosis in the threshold range of about 0.15–0.81, with a net benefit of up to 0.41

**Fig. 4:**
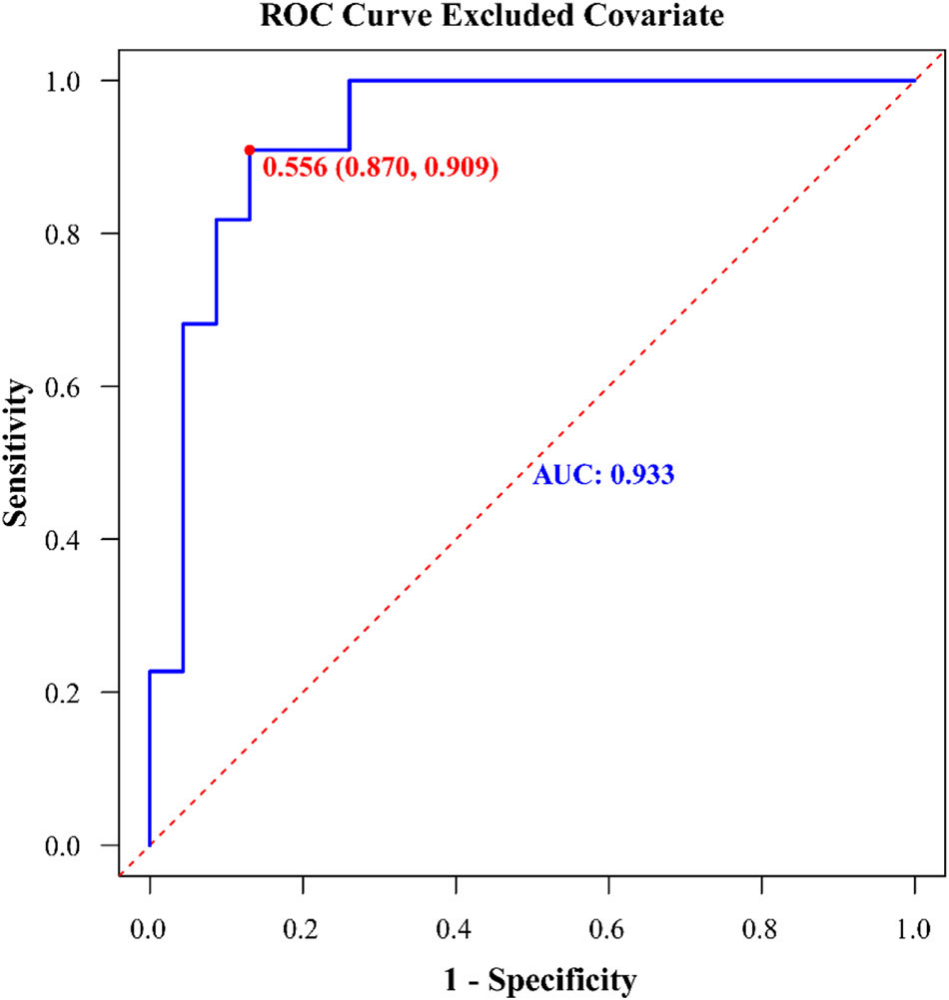
The ROC curve with inflammation as the covariable effector. The AUC is 0.933 (95% confidence interval: [0.856, 1.000], sensitivity is 90.9%, specificity is 87.0%)

Clinicians can directly input ED and HU_MonoE50keV_ values obtained during patient examinations into this dynamic web version to obtain the probabilities of moderate-severe fibrosis within specific segments of their diseased intestinal walls. Among them, the AUC of our training set for differentiating the none-mild fibrosis group from the moderate-severe fibrosis group was 0.828 (95% CI: 0.705–0.951). The sensitivity and specificity were determined to be 77.3% and 82.6%, respectively, while the C-index yielded consistent results (Fig. 3A). The calibration curve demonstrated a strong concordance between our nomogram model’s assessment of fibrosis within the training set and actual pathological findings (Fig. 3B). Furthermore, according to the DCA curve, our prediction model exhibited optimal performance in distinguishing these two groups when considering a threshold probability range of 0.15–0.81 from diseased intestinal wall (Fig. 3C). By validating the stability of the diagnostic model, we also controlled for the inflammation score of the affected intestinal wall as a covariate. As depicted in Fig. 4, upon eliminating the influence of inflammation, our model exhibited improved efficacy in fibrosis diagnosis, with an AUC of 0.933 (95% CI: 0.856–1), a sensitivity of 90.9%, and a specificity of 87.0%. However, no statistically significant difference was found according to the Delong test (*p* = 0.168).

**Fig. 5:**
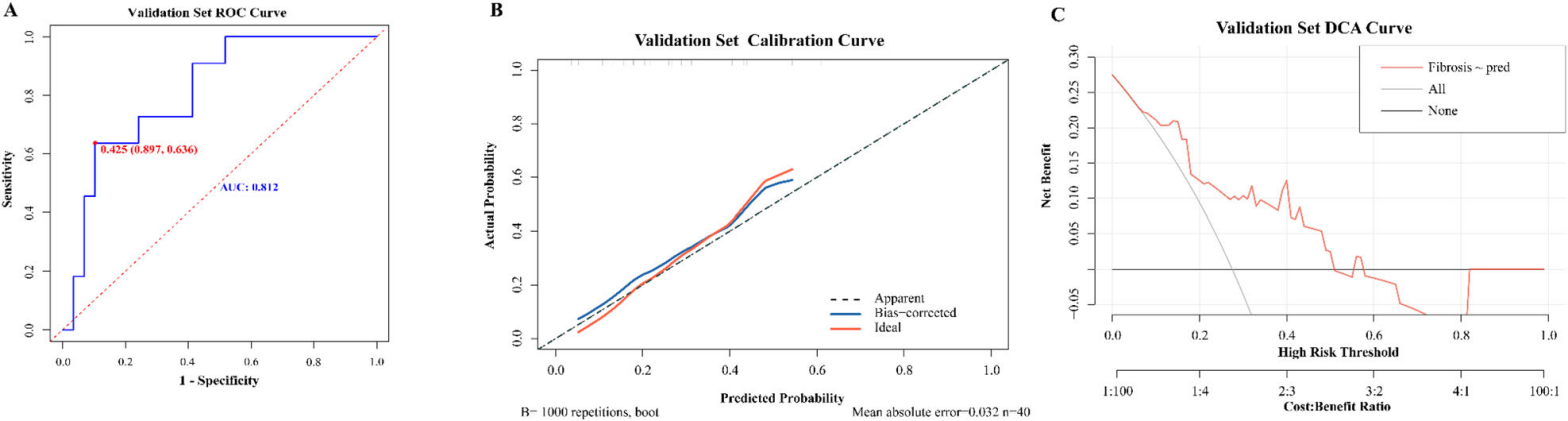
Analysis of the performance of fibrosis diagnostic model in validation set. **A** The ROC curve for the validation set data. The nomogram prediction model performs well in distinguishing CD intestinal wall None-mild fibrosis from Moderate-severe fibrosis in the validation set data, with an AUC of 0.812 (95% confidence interval: [0.676, 0.948], sensitivity is 63.6%, specificity is 89.7%) and a C-index of 0.812. **B** The ratio between the mean diagnostic probability of the nomogram model indicating the degree of fibrosis and the actual pathological outcomes in the validation set. A locally weighted regression line is plotted to show the overall trend. The red line represents the ideal curve. It shows that the nomogram prediction model has a good agreement between the prediction of the degree of intestinal wall fibrosis in Crohn’s patients and the actual pathological results in the validation set. **C** The DCA curve of nomogram diagnostic model in validation set. The curve shows that in the threshold range of about 0.08–0.50, the model provides a high benefit value for indicating fibrosis in patients with Crohn’s disease, with a net benefit of up to 0.22

The diagnostic performance of the dynamic nomogram model was validated using 40 intestinal specimens from six patients in the validation group. In this set, the model demonstrated an AUC of 0.812 (95% CI: 0.676–0.948), with a sensitivity of 63.6% and specificity of 89.7% (Fig. 5A). The C-index and calibration curves (Fig. 5B) also exhibited excellent agreement between the assessment of fibrosis in the validation set and the pathological fibrosis score (*p* > 0.05). Furthermore, DCA curves from the validation set revealed that our model had optimal gain in differentiating between none-mild and moderate-severe fibrosis groups when considering a threshold probability range of 0.08–0.50 for moderate-severe fibrosis (Fig. 5C).

### The 3D-printing technique enhanced the diagnostic efficiency of multi-parameters from spectral CT in validating intestinal fibrosis

**Fig. 6:**
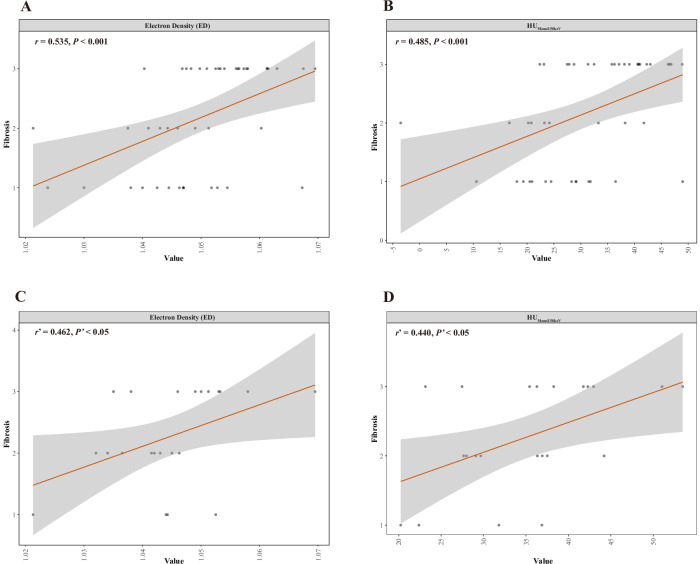
Comparison of the diagnostic efficiency of multi-parameters in spectral CT in validating intestinal fibrosis with or without 3D-printing technique. **A** and **B** are the results of with 3D-printing technique, while **C** and **D** are without 3D-printing technique. **A** and **B** show the correlation coefficient between ED and the degree of intestinal wall fibrosis is 0.53 (*p* < 0.001) and 0.49 (*p* < 0.001) for HU_MonoE50keV_; while it is 0.46 (*p* < 0.05) for ED and 0.44 (*p* < 0.05) for HU_MonoE50keV_, which is demonstrated in **C** and **D**

By employing random seeds, we randomly screened imaging parameters of the affected intestinal segment to form none-regional correspondences with intestinal wall specimens for subsequent correlation analysis with fibrosis scores. In the case of the model of none-3D printing, the correlation coefficients of ED, HU_MonoE50keV_, HU_MonoE70keV,_ HU_MonoE80keV_, and HU_MonoE110keV_ are lower compared to those observed in 3D printing, while no significant correlation was found between fibrosis and HU_MonoE40keV_, HU_MonoE60keV,_ HU_MonoE90keV_, HU_MonoE100keV_, HU_MonoE120keV_, or HU_MonoE140keV_ anymore (Table 5). However, the *p*-value between fibrosis score and HU_MonoE130keV_ increased from *p* = 0.002 to *p* < 0.001 when comparing none-3D printing with 3D-printing conditions with the correlation coefficient decreased relatively from 0.612 to 0.540. The results indicated that there was a stronger correlation between these parameters and fibrosis score in the case of 3D printing compared to none-3D printing technique (Fig. 6).

**Table 5:** Comparison of correlation analysis between parameters and fibrosis score in the model with 3D printing and without 3D printing technique (None-3D Printing)

Parameters	3D printing		None-3D printing	
	*r*	*p*-value	*r*^a^	*p*-value^a^
Z-Effective	0.096	0.531	0.061	0.789
ED	0.535	< 0.001*	0.462	0.030*
λ_1_	0.086	0.575	−0.015	0.948
λ_2_	0.088	0.565	−0.125	0.580
ΔHU_MonoE_	0.089	0.561	0.037	0.871
HU_MonoE40keV_	0.484	< 0.001*	0.158	0.483
HU_MonoE50keV_	0.485	< 0.001*	0.440	0.040*
HU_MonoE60keV_	0.540	< 0.001*	0.407	0.060
HU_MonoE70keV_	0.534	< 0.001*	0.462	0.030*
HU_MonoE80keV_	0.529	< 0.001*	0.429	0.046*
HU_MonoE90keV_	0.527	< 0.001*	0.414	0.055
HU_MonoE100keV_	0.531	< 0.001*	0.271	0.222
HU_MonoE110keV_	0.537	< 0.001*	0.440	0.040*
HU_MonoE120keV_	0.540	< 0.001*	0.260	0.242
HU_MonoE130keV_	0.540	< 0.001*	0.612	0.002*
HU_MonoE140keV_	0.534	< 0.001*	0.154	0.494

Compared with the situation of 3D printing, the correlation coefficients of ED, HU_MonoE50keV,_ HU_MonoE70keV_, HU_MonoE80keV_ and HU_MonoE110keV_ in None-3D printing are reduced; the correlation between HU_MonoE40keV_, HU_MonoE60keV_, HU_MonoE90keV_, HU_MonoE100keV_, HU_MonoE120keV_, HU_MonoE140keV_ and the degree of fibrosis is not obvious. The correlation coefficient between HU_MonoE130keV_ and intestinal fibrosis score increases

*ED* electron density, *HU* hounsfield unit

* *p* < 0.05; *r*, the correlation coefficient between the imaging parameters and the fibrosis score of the diseased intestinal wall in the case of with 3D-printing technique; *p*-value, *p*-value of correlation analysis between imaging parameters and fibrosis score in the case of with 3D-printing technique; *r*^a^, the correlation coefficient between imaging parameters and the fibrosis score of the lesion wall in the case of None-3D printing; *p*^a^ value, *p*-value of correlation analysis between imaging parameters and fibrosis score in the case of None-3D printing, λ, energy spectrum curve-slope; λ_1_, (HU_MonoE40keV_-HU_MonoE70keV_)/30; λ_2_, (HU_MonoE40keV_ -HU_MonoE140keV_)/100; ΔHU_MonoE_ = HU_MonoE40keV_ -HU_MonoE140keV_

## Discussion

Fibrosis plays a crucial role in the disability experienced by patients with CD, and accurate diagnosis of fibrosis serves as a pivotal factor for decision-making in CD management [32, 33].

The utilization of spectral CT offers a multitude of parameters for the diagnosis and quantification of fibrosis. Previous literature has extensively examined the benefits of spectral CT in evaluating intestinal lesions in patients with CD, including its ability to quantify disease activity, etc. However, there is currently a dearth of studies focusing on the analysis of intestinal fibrosis in CD patients [23, 34]. Based on the correlation between multi-parameter analysis and pathological results obtained from spectral CT, we have developed an evaluation model for diagnosing fibrosis in patients with CD. Our findings demonstrated that the AUC of the spectral CT parameter evaluation model for identifying intestinal fibrosis was 0.828 (95% CI: 0.705–0.951), with a sensitivity of 77.3% and specificity of 82.6%. In our previous study, we found that conventional contrast enhanced CT (CECT) assisted by artificial intelligence exhibited similar diagnostic performance to the spectral CT multiparameter model (AUC about 0.8) [24]. Building upon these findings, we have further established a web-based dynamic nomogram, which offers an effective and user-friendly technical approach for assessing fibrosis.

In addition, CD intestinal fibrosis exhibits significant spatial heterogeneity, making it challenging to comprehensively interpret the full extent of intestinal lesions through a single-layer pathological evaluation. In this study, we employed 3D-printing technique to establish a layer-to-layer correspondence between CT images and pathological analysis, enabling an accurate assessment of intestinal fibrosis. The results demonstrated that compared to none-3D printed imaging parameters, the layer-to-layer correspondence achieved stronger correlation with the pathological scores of intestinal lesions (Fig. 6). Turkbey et al used a customized 3D mold to register magnetic resonance imaging of the prostate and histopathological sections of specimens [25]. Another preclinical study evaluating the application efficiency of 3D-printing technique was conducted by Jardim-Perassi et al [26]. These aforementioned studies have substantiated the advantages of utilizing 3D-printing technique in disease diagnosis and treatment.

The accurate assessment of fibrosis in diseased intestinal segments is expected to provide precise information for surgical decision-making, thereby reducing unnecessary surgical resection and effectively improving patients’ quality of life.

Since inflammation and fibrosis often coexist in intestinal lesions in patients with CD [35, 36], we controlled for the inflammation score as a covariate to test the stability of the diagnostic model in differentiating fibrosis. The results demonstrated that after excluding the influence of inflammation, the diagnostic efficacy of the model for determining the degree of fibrosis in diseased intestinal walls improved clearly, with an AUC of 0.933 (95% CI: 0.856–1.000), a sensitivity of 90.9%, and a specificity of 87.0%. However, there was no significant difference in the diagnostic performance of the model before and after excluding inflammatory effects according to Delong’s test (*p* = 0.168). Additionally, previous studies have indicated that conventional CT imaging techniques are also inadequate for diagnosing fibrosis, with most studies focusing on using CT scans to quantify inflammatory bowel segments rather than fibrosis [19]. CD is a chronic disease characterized by recurring episodes [37] and requires ongoing adjustments based on disease progression. Cross-sectional imaging plays a crucial role in monitoring CD patients’ conditions [38], resulting in many patients undergoing repeated cross-sectional examinations throughout their lives. Our study also revealed that none-contrast spectral CT parameters exhibited a diagnostic performance of AUC with 0.828 for detecting fibrosis and could effectively reduce reliance on CECT scans, thereby minimizing radiation exposure and associated medical risks.

Our study had several advantages. The accurate co-registration between CT imaging and resected bowel specimens had been a challenging task. In this proof-of-concept study, we used 3D-printing technique to ensure optimal layer-to-layer alignment [25, 26]. To improve the accuracy of 3D printing-assisted localization, we carefully selected inflamed intestines with well-defined shapes and fixed positions across multiple cross-sectional imaging and minimized any noticeable deformation when in contact with specimens. Additionally, our study used continuous whole-circle intestinal specimens; a few previous studies [39] on CD imaging also used whole-circle specimens (though some had been dissected).

However, this study also had limitations. The sample size of patients was limited, which is common in proof-of-concept studies. To address this limitation, when testing the diagnostic efficacy of spectral CT in intestinal fibrosis, the Bootstrap method was employed with 1000 repetitions to estimate the statistics for calibration curves, to partially address dataset imbalance, as well as overcome limitations arising from small sample size and lack of external validation. Additionally, this study only focused on the terminal ileum, thus requiring further investigation to determine whether the findings could be extrapolated to other parts of the small intestine or colon.

In conclusion, the combination of multi-parameter spectral CT and 3D-printing technique seems to be able to assess the extent of intestinal fibrosis in Crohn’s disease. Although inflammation has an impact on evaluation efficiency, our diagnostic model is still effective in assessing the severity of fibrosis.

## Supplementary information

ELECTRONIC SUPPLEMENTARY MATERIAL

## Data Availability

The data that support the findings of this study are available from the corresponding author, Zhoulei Li (email: lizhlei3@mail.sysu.edu.cn), upon reasonable request. The data are not publicly available since this could compromise the privacy of research participants.

## Abbreviations

AUC: Area under the receiver operating characteristic curve
CD: Crohn’s disease
CECT: Contrast enhanced CT
CI: Confidence interval
CTE: Computed tomography enterography
ED: Electron density
HU: Hounsfield unit
ROC: Receiver operating characteristic
ROI: Region of interest
Spectral CT: Dual layer spectral CT
