# Supplementary material for: Intestinal fibrosis assessment in Crohn’s disease patient using unenhanced spectral CT combined with 3D-printing technique
Source: Insights Imaging. 2025 Mar 20;16:62. doi: 10.1186/s13244-025-01914-w (PMC11926292; doi:10.1186/s13244-025-01914-w)
Supplement: Supplementary file 1 — ELECTRONIC SUPPLEMENTARY MATERIAL [file 13244_2025_1914_MOESM1_ESM.pdf]

Intestinal fibrosis assessment in Crohn's disease patient using unenhanced spectral CT combined with 3D-printing-technique

ELECTRONIC SUPPLEMENTARY MATERIAL

Contents

Supplementary Materials 3

Supplementary Material 1. Gastrointestinal preparation and scanning protocol parameters involved in CTE scanning. 3

Supplementary Material 2. The procedural framework of 3D-printing technique for the co-registration of CTE images and intestinal specimens. 4

Supplementary Material 3. The details of statistical analysis. 8

Supplementary Material 4. The details of histological results. 10

Supplementary Material 5. The details of lasso regression. 11

Supplementary Material 6. The details of the nomogram. 12

Supplementary Tables 13

Supplementary Table 1. CTE scan parameters. 13

Supplementary Table 2. Images parameters*. 14

Supplementary Table 3. Histologic Scores for Intestinal Fibrosis and Inflammation. 15

Supplementary Table 4. Distribution of fibrosis score and inflammation score in diseased intestinal wall. 16

Supplementary Table 5. Difference analysis between correlation coefficient and partial correlation coefficient. 17

Supplementary Figure 18

Supplementary Figure 1. The ridge map. 18

Supplementary Figure 2. Radiomic nomograms for indication of fibrosis degree of CD patients.. 19

Supplementary Materials

Supplementary Material 1. Gastrointestinal preparation and scanning protocol parameters involved in CTE scanning.

After bowel preparation, 1600 to 2000ml of a 2.5% mannitol solution was administered for participants within one hour prior to the CT enterography (CTE) examination.

CTE examination was performed using the IQon Spectral CT system (Philips Healthcare, Nederland B.V.). The tube voltage was set at 120 kVp with DoseRight automatic tube current modulation technology. Data acquisition was conducted using the spiral volume scanning mode, with a pitch of 0.981 and rotation time of 0.5 seconds. The layer thickness is set to 1.0mm, with a spacing of 0.8mm. The reconstruction mode employed is iDose4, while the filtering algorithm utilizes the standard (B). The scanning protocol included a non-contrast scan, arterial phase (28 S), and venous phase (70 S).

Additionally, immediate *ex vivo* CT scans were conducted on the resected intestinal specimens from the participants post-surgery in order to ascertain their morphological characteristics.

The CTE scan and images parameters for participants and intestinal specimens are detailed in the **Supplementary Table 1-2**.

Supplementary Material 2. The procedural framework of 3D-printing technique for the co-registration of CTE images and intestinal specimens.

The longitudinal co-registration between CTE images and both intestinal specimens, as well as pathological sections, was achieved using 3D printing technique. The sequential steps were executed in the following manner:

A'. **Surgical procedures and *ex vivo* CTE scans of the targeted intestinal segment:** For the training set, based on the information obtained from venous phase CTE images, which were interpreted by both radiologists and surgeons to determine the targeted resected intestinal segment, The surgeon meticulously removes peristaltic fat surrounding the target intestinal segment, followed by staining and marking with green for the upper and lower boundaries. Subsequently, a radiologist promptly conducted an *ex vivo* CT scan (**Supplementary Material 1 and Supplementary Table 1-2**) to acquire additional *ex vivo* information, such as identifying the narrowest layer for surface localization.

A. **The targeted intestinal segment's volume of interest is delineated in 3D data:** For the validation set, the venous CTE images were initially imported into VitreaWorkStation (version 6.5.5; Vital Images, Inc; 5850 Opus Parkway, Suite 300; Minnetonka, MN, US; 55343). Subsequently, we meticulously delineated a three-dimensional volume of interest along the contour of the targeted intestinal segment on venous phase CTE images (red zone), utilizing information obtained from CTE interpreted by both

radiologists and surgeons. The resulting data was saved as STL format.

B. **Designing a 3D printed mold and revolving digital molds into physical objects through 3D printing technology:** The aforementioned 3D data (STL) were imported into AutoDesk Fusion360 (version 2.0.16490; Autodesk Asia Pte. Ltd., 3 Fusionopolis Way, #10-21 Symbiosis, Singapore 138633) for the purpose of reconstructing the 3D model of the targeted segment of the intestine. Subsequently, a 3D printing model was designed based on this model. The dimensions and configuration of the cavity within the 3D printing model used to contain the surgically excised specimen were consistent with those observed in venous phase images, accurately representing the targeted intestinal segment for validation set. For training set the mold was designed directly based on the spectral CT imaging from the resected intestines. The mold incorporates multiple grooves with a width of 2 mm and a spacing of 3 mm, which were determined based on CTE imaging parameters such as slicing position, thickness, and spacing. Specifically, the grooves are positioned with a center-to-center spacing of 5 mm (equivalent to the CTE image slice spacing), and the quantity of grooves is determined by the length of the target intestine (The model depicted in figure **B** exhibits a total of 7 grooves, with the 4th groove positioned at the center. The spacing between each groove measures 5mm). These slots were specifically designed to facilitate the precise slicing of the intestine for generating slices, corresponded accurately to those

obtained from CTE imaging. Then, utilizing Bambu Lab's 3D printing equipment (Bambu Lab version X1E; Bambu Lab, Guoxin Investment Building, Nanshan District, Shenzhen, Guangdong Province) to transform the model into a physical mold.

C. **Performing surgical procedures and repositioning the intestinal specimen inside a 3D printing mold:** For validation set, based on the information obtained from venous phase CTE images, which were interpreted by both radiologists and surgeons to determine the targeted resected intestinal segment, the surgeon meticulously removes peristaltic fat surrounding the target intestinal segment, followed by staining and marking with green for the upper and lower boundaries. All intestinal specimens (both training and validation sets) were placed inside the 3D printed mold using previously obtained positioning marks.

D. ***Ex vivo* CTE scans of the targeted intestinal segment:** For the validation set, in order to obtain additional ex vivo information, such as identifying the narrowest layer for surface localization, a radiologist promptly conducted an ex vivo CT scan (**Supplementary Material 1 and Supplementary Table 1-2**).

E. **Specimen sectioning:** The intestinal specimen was dissected along the designated slots to obtain axial whole-circle sections of the intestine. The obtained sections were further divided into subsections that were 4 μm thick.

F. **Pathological imaging of the intestinal specimen:** The intestinal sections were stained with Masson trichrome and Hematoxylin-eosin. Subsequently, the stained pathological samples were digitally scanned using PANNORAMIC DESK (3DHISTECH Ltd; H-1141 Budapest, Öv u. 3, Hungary) to obtain electronic whole-slide images. CaseViewer (version 2.4; 3DHISTECH Ltd.; H-1141 Budapest, Öv u. 3, Hungary) was used for histopathological analysis (e.g., the 4th slice).

Supplementary Material 3. The details of statistical analysis.

The statistical analyses were conducted using SPSS version 26.0 software (IBM SPSS Statistics 26. Ink), Python, and R software (version 4.2.3). Two-sided comparisons were employed for the statistical analysis, with significance defined as a P<0.05. Quantitative data were presented as mean ± standard deviation or median (interquartile range), while qualitative data were expressed as percentages. We first used the Shapiro-Wilke Test to test the normality of each parameter. And then the bivariate correlation between CTE parameters and histological scores was analyzed using Spearman's rank correlation, while the partial correlation analysis was conducted after controlling for inflammation as a covariate. Furthermore, the differences in Z-Effective, ED, $HU_{MonoE40\text{-}140keV}$, energy spectrum curve-slope λ1, λ2, and ΔHU_{MonoE} between different histological grades were assessed using two independent samples t-test or Man-Whitney U test, depending on whether the parameters conform to a normal distribution.

In the subsequent multivariable logistic model analysis, the image parameters associated with fibrosis identified through lasso analysis were included. A dynamic nomogram of the model was constructed with ED and $HU_{MonoE50keV}$ to supply a convenient and free using method for diagnosis of intestinal fibrosis. The differentiation ability and calibration performance of the dynamic nomogram of the model was evaluated by generating calibration curves, DCA diagrams, and receiver operating characteristic (ROC) curves.

The area under the ROC curve (AUC) value was calculated to determine the diagnostic accuracy of CTE parameters for intestinal fibrosis. During the validation model, we calculated the AUC, sensitivity and specificity for intestinal fibrosis diagnosis. Bootstrap method has been applied to improve the stability of the findings and reduces the potential impact of random error. Finally, by employing random seeds, we randomly screened imaging parameters of the affected intestinal segment while ensuring reproducibility to achieve a normal corresponding relation of pathology to imaging in clinical routine, i.e., without utilizing 3D printing technique. We then compared the diagnostic performance of fibrosis between 3D printing and none-3D printing conditions.

Supplementary Material 4. The details of histological results.

The diseased intestinal wall with a total of 85 segments were classified into four categories based on the degree of pathological fibrosis under score from 0 to 4 (**Supplementary Table 3**). Similarly, lesions were categorized with 4 degrees based on the severity of inflammation observed in the same segments (**Supplementary Table 3**). It is worth noting that higher degrees of inflammation and fibrosis are often associated with an increased risk of intestinal stricture as well as surgery. To better identify patients at risk for surgery, we defined the none-mild fibrosis group as those with a fibrosis score ranging from 0 to 2, which accounted for approximately 61.18% of all cases (52 out of 85); while the moderate-severe fibrosis group included individuals with a fibrosis score ranging from 3 to 4 (38.82%, 33 out of 85). Similarly, we defined the none-mild inflammatory group as those with an inflammatory score ranging from 0 to 2 (41 out of 85); whereas individuals with an inflammatory score ranging from 3 to 4 were classified under the moderate-severe inflammatory subgroup (44 out of 85) (**Supplementary Table 3-4**).

Supplementary Material 5. The details of lasso regression.

The LASSO regression screening process adhered to the prescribed formula：

$$J(\beta) = \sum(y - X\beta)^2 + \lambda \parallel \beta \parallel_1$$

$\sum(y - X\beta)^2$ represented the sum of squares of the error, $X\beta$ is the predicted value obtained by the regression model, $\lambda \parallel \beta \parallel_1$ represented the regular term, λ corresponds to the λ in **Figure 2** for selecting an appropriate weight for the regular term, $\parallel \beta \parallel_1$ denoted the L1 norm.

Supplementary Material 6. The details of the nomogram.

We then divided 85 intestinal samples into a training set (6 patients, 45 samples) and a validation set (6 patients, 40 samples). Subsequently, we constructed a radiomics model based on the two selected parameters and developed a radiomics nomogram (**Supplementary Figure 2A**). By utilizing the patient's ED and $HU_{MonoE50keV}$ values, the total score of the model can be calculated to predict the probability of moderate-severe fibrosis in CD patients' using the corresponding nomogram. Additionally, for enhanced convenience in assessing fibrosis based on CTE parameters, we established a web-based dynamic nomogram accessible at ***https://dynanomo.shinyapps.io/Fibrosis_Prediction/*** (**Supplementary Figure 2B**) by using the R software.

Supplementary Tables

Supplementary Table 1. CTE scan parameters.

Scan parameters	non-contrast*	Arterial phase	Venous phase
Tube Voltage(kVp)	120	120	120
Tube Current (DoseRight mode)	Index:24	Index:24	Index:24
Pitch	0.891	0.891	0.891
Rotation Time(s)	0.5	0.5	0.5
Scan mode	Helical	Helical	Helical

Note. *It is also used for scanning *ex vivo* intestinal specimens.

Supplementary Table 2. Images parameters*.

Parameters	$HU_{MonoE40-140keV}$	Z Effective	Electron Density
Thickness(mm)	1.0	1.0	1.0
Increment (mm)	0.8	0.8	0.8
Reconstruction model	$iDose^4$	$iDose^4$	$iDose^4$
Filter	Standard(B)	Standard(B)	Standard(B)

Note. * Derived from the spectral-based image (SBI) of non-contrast phase.

Supplementary Table 3. Histologic Scores for Intestinal Fibrosis and Inflammation.

Score	Fibrosis	Inflammation
None-mild disease		
0	No fibrosis	No inflammation or distortion
1	Minimal fibrosis in submucosa or subserosa	Lamina propria inflammation only
2	Increased submucosal fibrosis, septa into muscularis propria	Submucosal foci of inflammation
Moderate-severe disease		
3	Septa through muscularis propria, increase in subserosal collagen	Foci of transmural inflammation
4	Significant transmural scar, marked subserosal collagen	Significant, dissecting, confluent transmural inflammation

Supplementary Table 4. Distribution of fibrosis score and inflammation score in diseased intestinal wall.

	Fibrosis					Inflammation				
	0	1	2	3/4	Total	0	1	2	3/4	Total
Training Set	0	14	9	22	45	2	0	17	26	45
Validation Set	3	11	15	11	40	2	10	10	18	40
Total	3	25	24	33	85	4	10	27	44	85

Supplementary Table 5. Difference analysis between correlation coefficient and partial correlation coefficient.

Parameters	*r*	*z*	*γ*	*z'*	*d*	*SE*	*z* value	*P* value
Z Effective	0.096	0.096	0.046	0.046	0.050	0.218	0.230	0.818
ED	0.535	0.597	0.605	0.701	-0.104	0.218	-0.476	0.634
λ_1	0.086	0.086	0.005	0.005	0.081	0.218	0.372	0.709
λ_2	0.088	0.088	0.003	0.003	0.085	0.218	0.391	0.696
ΔHU_{MonoE}	0.089	0.089	0.003	0.003	0.086	0.218	0.395	0.693
$HU_{MonoE40keV}$	0.484	0.528	0.475	0.517	0.012	0.218	0.054	0.957
$HU_{MonoE50keV}$	0.485	0.530	0.524	0.582	-0.052	0.218	-0.240	0.810
$HU_{MonoE60keV}$	0.540	0.604	0.550	0.618	-0.014	0.218	-0.065	0.948
$HU_{MonoE70keV}$	0.534	0.596	0.563	0.637	-0.041	0.218	-0.190	0.849
$HU_{MonoE80keV}$	0.529	0.589	0.570	0.648	-0.059	0.218	-0.269	0.788
$HU_{MonoE90keV}$	0.527	0.586	0.574	0.653	-0.067	0.218	-0.309	0.757
$HU_{MonoE100keV}$	0.531	0.592	0.576	0.656	-0.065	0.218	-0.297	0.766
$HU_{MonoE110keV}$	0.537	0.600	0.578	0.659	-0.060	0.218	-0.273	0.785
$HU_{MonoE120keV}$	0.540	0.604	0.578	0.659	-0.055	0.218	-0.253	0.800
$HU_{MonoE130keV}$	0.540	0.604	0.580	0.662	-0.058	0.218	-0.267	0.789
$HU_{MonoE140keV}$	0.534	0.596	0.580	0.662	-0.067	0.218	-0.306	0.760

Note. As is shown in the **Supplementary Table 5**, there is no statistical difference between the correlation coefficient *r* and partial correlation coefficient *γ* ($P>0.05$).

r, the correlation coefficient between the imaging parameters and the fibrosis score of the diseased intestinal wall; *z*, the normal distribution value of the correlation coefficient obtained by Fisher transformation; *γ*, the partial correlation coefficient between imaging parameters and the fibrosis score of the lesions; *z'*, the normal distribution value of the partial correlation coefficient obtained by Fisher transformation; *d*, the difference value between *z* and *z'*; *SE*, the standard error of the difference; *z* value, the statistic corresponding to the normal distribution; *P* value, the corresponding bilateral *P* values in the standard normal distribution table; ED, electron density; λ, energy spectrum curve-slope; HU, hounsfield unit.

Supplementary Figure

Supplementary Figure 1. The ridge map.

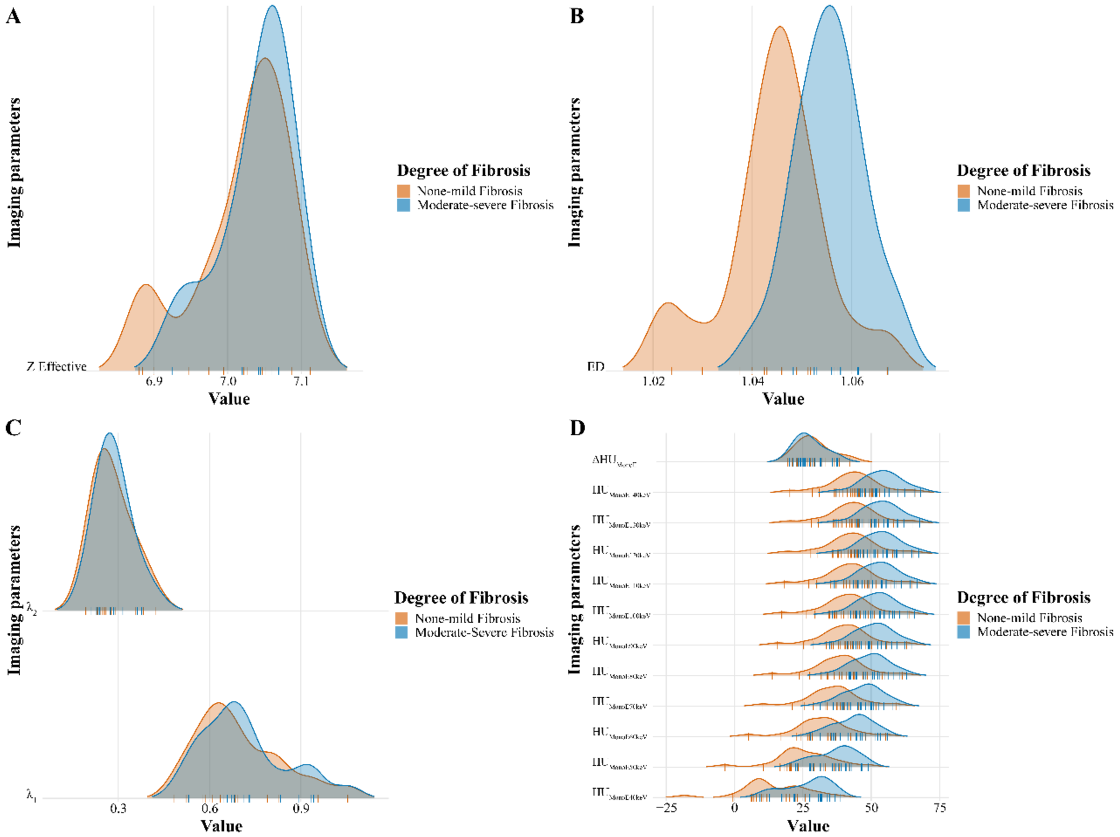

Supplementary Figure 1. The ridge map shows the numerical distribution of different imaging parameters in the group with none-mild fibrosis and the group with moderate-severe fibrosis in the training set.

Supplementary Figure 2. Radiomic nomograms for indication of fibrosis degree of CD patients.

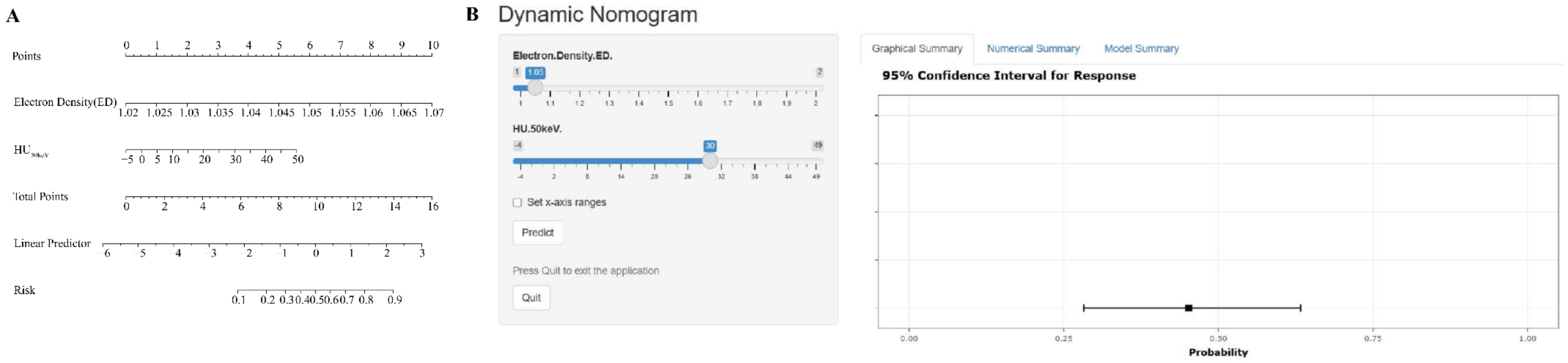

Supplementary Figure 2. (A) A nomogram in the training set by including the following two parameters: ED and $HU_{MonoE50keV}$. A fibrosis score of 0-2 is defined as None-mild fibrosis and assigns as 0. A score of 3-4 is defined as Moderate-severe fibrosis and assigns as 1. **(B)** Online dynamic nomogram: *https://dynanomo.shinyapps.io/Fibrosis_Prediction/*. It depicts an example for predicting the degree of intestinal wall fibrosis in patients with CD at ED of 1.05 and $HU_{MonoE50keV}$ of 30 in spectral CT.
